# Three Aromatic Residues are Required for Electron Transfer during Iron Mineralization in Bacterioferritin

**DOI:** 10.1002/anie.201507486

**Published:** 2015-10-16

**Authors:** Justin M Bradley, Dimitri A Svistunenko, Tamara L Lawson, Andrew M Hemmings, Geoffrey R Moore, Nick E Le Brun

**Affiliations:** Centre for Molecular and Structural Biochemistry, School of Chemistry, University of East Anglia Norwich Research Park, Norwich, NR4 7TJ (UK) E-mail: n.le-brun@uea.ac.uk; School of Biological Sciences, University of Essex Wivenhoe Park, Colchester CO4 3SQ (UK); School of Biological Sciences Norwich Research Park University of East Anglia Norwich NR4 7TJ (UK)

**Keywords:** bioinorganic chemistry, ferritin, iron, mineralization, tyrosyl radicals

## Abstract

Ferritins are iron storage proteins that overcome the problems of toxicity and poor bioavailability of iron by catalyzing iron oxidation and mineralization through the activity of a diiron ferroxidase site. Unlike in other ferritins, the oxidized di-Fe^3+^ site of Escherichia coli bacterioferritin (EcBFR) is stable and therefore does not function as a conduit for the transfer of Fe^3+^ into the storage cavity, but instead acts as a true catalytic cofactor that cycles its oxidation state while driving Fe^2+^ oxidation in the cavity. Herein, we demonstrate that EcBFR mineralization depends on three aromatic residues near the diiron site, Tyr25, Tyr58, and Trp133, and that a transient radical is formed on Tyr25. The data indicate that the aromatic residues, together with a previously identified inner surface iron site, promote mineralization by ensuring the simultaneous delivery of two electrons, derived from Fe^2+^ oxidation in the BFR cavity, to the di-ferric catalytic site for safe reduction of O_2_.

The capacity of cells to store iron in a bioavailable and safe form is a central feature of the strategy nature has evolved to overcome the dual problem of poor bioavailability of iron and the potential of the free metal to catalyze the formation of reactive radical species. This function is fulfilled by the ubiquitous ferritin family, members of which are composed of 24 subunits arranged to form a protein shell surrounding a hollow center (Figure 1 a). Large amounts of iron can be stored within the central cavity, in the form of a ferric-oxy-hydroxide mineral.[1] Animal ferritins are commonly composed of a mixture of H-chains, which contain a catalytic dinuclear iron site known as the ferroxidase site, and l-chains, which lack the ferroxidase site. Prokaryotic and plant ferritins are composed exclusively of H-chain-like subunits. The H-chain ferroxidase sites of animal ferritins play a central role in the mineralization process, which involves binding of Fe^2+^, oxidation to Fe^3+^, and labilization/hydration to generate the final mineral form in the cavity.[2]

**Figure 1:**
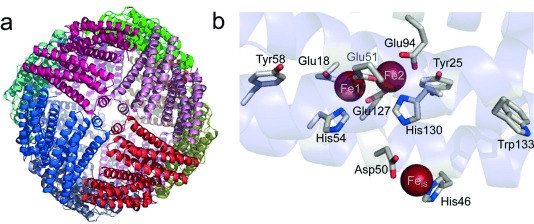
Structural features of EcBFR. a) Ribbon representation of the 24 meric ferritin viewed down one of six four-fold axes. b) The diiron ferroxidase site of BFR is shown with coordinating residues (Glu18 and His54 are terminal ligands to Fe1; Glu94 and His130 are terminal ligands to Fe2; Glu51 and Glu127 bridge Fe1 and Fe2), along with the inner surface iron site (Fe_IS_) with coordinating residues (His46 and Asp50), and closely lying aromatic residues (Tyr25, Tyr58, and Trp133). Generated using PyMol with PDB file 3E1M.[3c]

Bacterioferritins (BFRs) constitute a distinct sub-family of ferritins, found only in bacteria, which contain a markedly different diiron ferroxidase site (Figure 1 b).[3] The ferroxidase site of *E.* *coli* BFR (EcBFR) was previously shown to be stable in its oxidized di-Fe^3+^ form and essential throughout mineralization.[3c, 4] This led to a proposed mechanism in which the ferroxidase site functions as a true catalytic center, continually cycling between its oxidized (bridged di-Fe^3+^) and reduced (di-Fe^2+^) forms, and driving oxidation of Fe^2+^ ions in the central cavity and O_2_ reduction at the diiron site.[5] The model requires the existence of an electron transfer route from the cavity to the ferroxidase site. An Fe^2+^-binding site located on the inner surface of the subunit, ∼10 Å away from the ferroxidase site and coordinated by His46, Asp50, and three water molecules, was subsequently identified.[3c] Disruption of this site severely inhibited mineralization, consistent with an important role in electron transfer from Fe^2+^ ions in the cavity to the ferroxidase site. However, until now direct evidence for electron transfer was missing.

The EcBFR ferroxidase site is flanked by two Tyr residues (Tyr25 and Tyr58) that are ∼4.0–6.5 Å away from the nearest iron, and by a Trp residue (Trp133), which is ∼9.7 Å away from site FeB (Figure 1 b). BFR also contains five other Tyr and one other Trp, which are located further from the ferroxidase site (Supporting Information, Figure S1). We sought to determine whether these aromatic residues are involved in coupling electron transfer to oxidation of Fe^2+^. Site-directed variants, in which each residue was substituted with Phe, were generated and the mineralization activity of the variant proteins was determined. Figure 2 shows changes in absorbance at 340 nm upon the addition of 400 Fe^2+^ ions per protein. Substitutions of Tyr25, Tyr58, and Trp133 resulted in significantly slower mineralization, with rates of 15 % (Y25F), 30 % (Y58F), and 25 % (W133F) relative to wild type BFR (Figure 2 a). Y45F exhibited an initial rate 50 % that of wild type BFR. Rates for the five other Tyr/Trp variants were 70 % (W35F, Y10F, Y107F), 75 % (Y114F) and 85 % (Y149F) that of wild type BFR (Figure 2 b).

**Figure 2:**
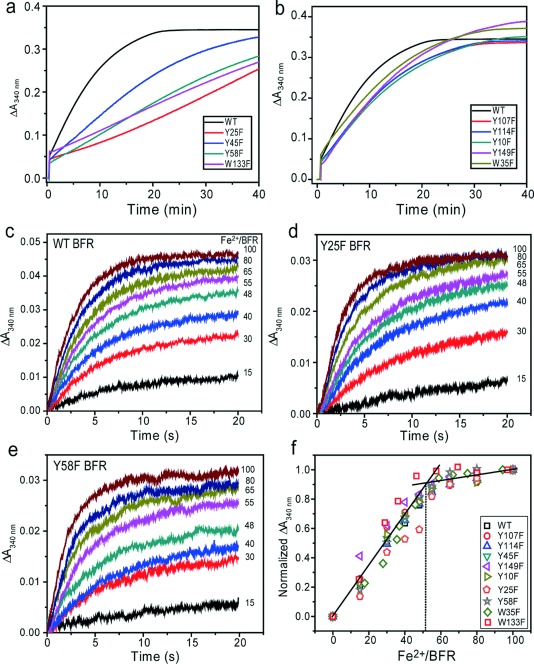
Kinetics of Fe^2+^ oxidation in EcBFR aromatic residue variants. a, b) ΔA_340 nm_ as a function of time following aerobic additions of 400 Fe^2+^/protein to wild type BFR and variant, as indicated. c-e) ΔA_340 nm_ measured by stopped-flow following additions of 0–100 Fe^2+^ per wild type (c), Y25F (d), and Y58F (e) BFR. The proteins (0.5 μm) were in 0.1 m MES pH 6.5. Temperature was 25 °C and path length 1 cm. f) Plot of normalized ΔA_340 nm_ for the initial ferroxidase site reaction as a function of the Fe^2+^/protein ratio, as indicated. Saturation of rapid oxidation is indicated by the intersection of initial and final linear regions of the plots. Data points are from (c–e) and the Supporting Information, Figure S2.

As ferroxidase site activity is essential for mineralization in BFR,[3a, 5] we investigated whether the severely slowed mineralization in variants Y25F, Y58F, and W133F was due to impaired initial oxidation of Fe^2+^ at the ferroxidase site. Stopped-flow absorbance spectroscopy was used to monitor changes following the aerobic addition of Fe^2+^ to EcBFR and variants. In all of these cases, the variants were very similar to wild type BFR (Figure 2 c–e; Supporting Information, Figure S2),[6] demonstrating that the initial oxidation of Fe^2+^ at the ferroxidase site of the variant proteins is essentially unaffected. Furthermore, plots of Δ*A*_340 nm_ as a function of Fe^2+^ ions per BFR (Figure 2 f) showed clearly that the initial rapid oxidation phase saturates at two Fe^2+^ ions per subunit (∼50 per 24 mer) in each variant, as observed for the wild type protein.[4a]

X-ray crystal structures were determined for the Y45F, Y114F, and Y149F variants (Supporting Information, Table S1, Figure S3). These, along with previously reported structures of W35F and W133F BFRs[6] and Y25F and Y58F BFR subunit dimers,[7] showed that the substitutions did not cause any significant changes beyond the substituted side chain. Because these residues are not required for, nor affect, the initial binding or oxidation of Fe^2+^ at the ferroxidase site, Tyr25, Tyr58, and Trp133 must have important functional role(s) in mineralization only after the initial formation of the di-ferric ferroxidase site. We note that mineralization was not entirely abolished in the Y25F, Y58F, and W133F variants. Ferritins catalyze Fe^2+^ oxidation, a thermodynamically favorable reaction, and so inhibition of ferritin function through the substitution of a functionally important residue can never result in complete loss of Fe^2+^ oxidation.

In some cases, oxidation of aromatic residues involved in electron transfer can be detected. Thus, EPR spectroscopy was used to investigate if a radical species is formed during iron mineralization. Fe^2+^ was added aerobically to apo-BFR (at a loading of 72 per protein, chosen because the aromatic residues only become functionally important above 48 Fe^2+^ per protein) and the sample was frozen after ∼10 s. The resulting EPR spectrum contained a signal in the *g*=2 region that was not present prior to the addition of Fe^2+^ (Figure 3 a). The signal was simulated as a tyrosyl radical using parameters derived from the TRSSA program,[8] with Tyr ring rotation angle *θ*=−17° and C1 atom spin density *ρ*_c1_=0.408 (Figure 3 a). The complete set of simulation parameters are given in Table S2 (Supporting Information).

**Figure 3:**
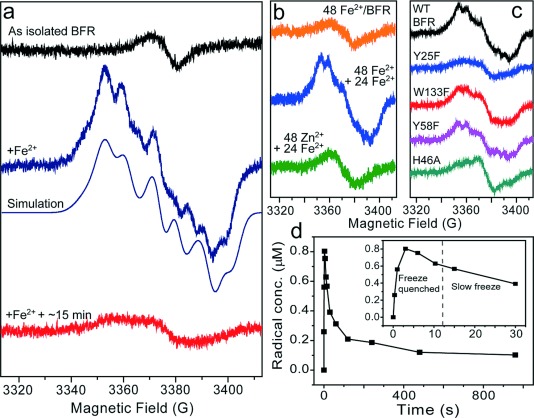
EPR analysis of a tyrosyl radical in BFR. a) EPR spectra of wild type BFR as isolated and following the addition of 72 Fe^2+^ per protein, as indicated. The Tyr radical spectrum was simulated with the parameters found by TRSSA[8] and reported in the Supporting Information, Table S2. The lower spectrum was recorded 15 min after the addition of Fe^2+^, showing that the radical signal decays. b) EPR spectra of: wild type BFR following the aerobic addition of 48 Fe^2+^/protein and 10 min incubation (upper); the same sample (containing 48 Fe^3+^) following the further addition of 24 Fe^2+^ per protein (middle); wild-type BFR containing 48 Zn^2+^ ions per protein following the addition of 24 Fe^2+^ ions. c) EPR spectra following addition of 72 Fe^2+^ per protein to wild type BFR and variants, as indicated. d) Plot of Tyr25 radical concentration in wild type BFR after addition of 72 Fe^2+^ per protein in the range of 0.3 s–16 min. The plot is a result of merging two data sets obtained for rapidly freeze-quenched and standard frozen samples (Supporting Information, Figure S5a, b). A correction factor (2.1) was applied to the freeze-quenched data to account for a systematic difference in the two methods, which most likely originates from a very different ratio of mixing volumes. For all EPR experiments BFR was 8.3 μm in 0.1 m MES pH 6.5, and spectra were obtained at 10 K at the following instrumental conditions: microwave frequency *ν*=9.467 GHz, microwave power P_MW_=0.05 mW, modulation frequency *ν*_M_=100 kHz, modulation amplitude *A*_m_=3 G, scan rate *v*=0.596 G s^−1^, time constant *τ*=82 ms. In (a–c), solutions were frozen within 10 s of iron addition unless otherwise indicated.

To confirm that the radical is formed principally after the initial oxidation of Fe^2+^ at the ferroxidase sites, 48 Fe^2+^/BFR were added aerobically and allowed to fully oxidize. The EPR spectrum (Figure 3 b) showed no evidence of the tyrosyl radical. Addition of a further 24 Fe^2+^ ions resulted in formation of the radical (Figure 3 b), and we conclude that radical formation is indeed linked to the aromatic residues being functionally important in mineralization. Pre-incubation of EcBFR with Zn^2+^ (at 48/protein), a potent inhibitor of mineralization that binds tightly at the ferroxidase sites,[3a],[c] did not result in radical formation (Figure 3 b). The data demonstrate that the ferroxidase site is required for formation of the Tyr radical, but only after the initial oxidation of Fe^2+^ at the ferroxidase site.

EPR studies of the EcBFR variants revealed that Tyr25 is the site of radical formation; only in Y25F was the Tyr radical not observed (Figure 3 c; Supporting Information, Figure S4). This agrees well with a conformational analysis of all of the Tyr side-chains in six structures of wild type BFR (Supporting Information, Figure S5), which revealed that Tyr25 is the only Tyr residue that has been structurally characterized in the predicted radical conformation, and is the only Tyr residue that is conformationally flexible. The concentration of the radical was low, ∼1 % of subunit concentration (∼24 % of protein concentration), and so it is possible that it results from a minor, off-pathway reaction. However, because Tyr25 is functionally important, it is more likely that the observation of a radical is important for understanding the role Tyr25 plays in mineralization. The radical would have to be transiently formed, and evidence for this is shown in Figure 3 a,d (and Supporting Information, Figure S6); the signal formed rapidly in seconds and decayed in minutes following completion of Fe^2+^ oxidation when no further Fe^2+^ is available to quench the remaining Tyr radicals. During mineralization, when Fe^2+^ is available, quenching of the radical would be expected to occur much more rapidly, at a rate similar to that of radical formation.

The inhibition of mineralization observed in the three near-ferroxidase site variants was very similar to that observed previously for inner surface site variants H46A and D50A,[3c] indicating that the inner surface site and aromatic residues are involved in the same process. Consistent with this, EPR measurements of H46A showed that the Tyr25 radical is not formed in the absence of a functional inner surface site (Figure 3 c).

The data presented here support a model for mineralization in which the ferroxidase site functions as a true catalytic center, continually cycling between its oxidized (di-Fe^3+^) and reduced (di-Fe^2+^) states.[3c, 5] Cycling of the site is driven by the oxidation of Fe^2+^ in the central cavity, with resulting electrons channeled to the ferroxidase site causing reduction to the di-Fe^2+^ form, which is then primed to react again with O_2_ (or H_2_O_2_). Hydrolysis of the accumulating hydrated Fe^3+^ in the cavity leads to mineral formation. The importance of aromatic residues for mineralization, and the detection of a transient Tyr25 radical, provides important evidence about the origins of the two electrons required to re-reduce the ferroxidase site. We propose that one electron comes from the oxidation of the inner surface site Fe^2+^, while the other is from Tyr25, generating the radical. This is subsequently quenched by an electron that must be ultimately derived from a second Fe^2+^ (in EcBFR, all electrons needed for O_2_ reduction are derived from Fe^2+^),[4b] most likely in the cavity. The net effect of this is that two cavity Fe^2+^ ions are oxidized with the delivery of two electrons to the di-Fe^3+^ ferroxidase site. This enables near simultaneous arrival of the two electrons at the oxidized ferroxidase site (Figure 4), minimizing the possibility of single electron reduction of O_2_ or H_2_O_2_ at the site, and the accidental release of toxic reactive oxygen species. Consistent with this, in some cases, BFRs have been shown to have important functions in controlling oxidative stress in combination with iron storage.[9a–c]

**Figure 4:**
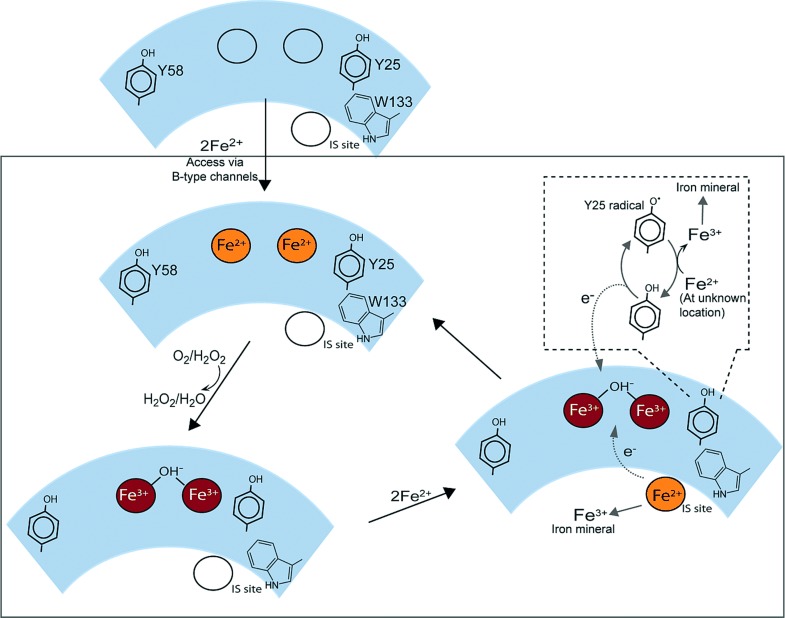
Proposed mechanism of mineralization in BFR. Two Fe^2+^ ions access the ferroxidase site through B-type channels[14] and undergo oxidation to the bridged di-ferric form; the site is now primed for its catalytic cycle. This involves binding and oxidation of Fe^2+^ at the inner surface site (IS site). A second electron is derived from the oxidation of the nearby Tyr25 side chain, generating a radical. The radical decays through an unknown mechanism involving oxidation of a second Fe^2+^. This occurs readily, ensuring that the reactive radical does not accumulate. Previous data indicated that iron oxidized at the inner surface site does not remain there,[3] and we propose that it nucleates or is incorporated into the growing mineral core. The now reduced diiron site is re-oxidized by O_2_ or H_2_O_2_, returning to its resting state.

Tyr25 is absolutely conserved in all 24 mer ferritins, and tyrosyl radical formation at this residue has been observed in other ferritins, including human H-chain,[10a] *E.* *coli* FtnA,[10b] and *Pyroccocus furiosus* Ftn,[10c] and it is reasonable to propose that Tyr radical formation is a common feature of ferritins. Aromatic residue radical formation was also observed during Fe^2+^ oxidation in the related Dps ferritins, pointing to a wider significance of radical chemistry in iron storage/detoxification proteins.[10d] The roles of aromatic radicals in iron mineralization are, in some cases, unclear but appear to vary between ferritins. In HuHF, radical formation on Tyr34 did not appear to be mechanistically important, because a Y34F variant was not significantly affected in its overall capacity to mineralize Fe^2+^.[10a] In *P.* *furiosus* Ftn, Tyr24 was found to be essential for the ferroxidase site oxidation reaction, and therefore mineralization,[10b] while in *E.* *coli* FtnA, Tyr24 was found to be important for mineralization but not the initial ferroxidase site reaction,[10c] as found here for BFR. This undoubtedly reflects the structural and mechanistic variation that exists amongst the ferritins.[11]

The R2 subunit of ribonucleotide reductase contains a diiron site closely related to that of BFR and generates a stable radical at the nearby Tyr122 (*E.* *coli* R2 numbering). The radical character is subsequently shuttled back and forth over >30 Å to the site of ribonucleotide reduction in the R1 subunit.[12] Tyr25 and Trp133 of BFR are in very similar positions to Tyr122 and Trp48 of R2, but on the other side of the diiron site. Trp48 of R2 has been proposed to be important for the radical transfer pathway.[13] The mechanism proposed here does not explicitly account for the importance of Tyr58 and Trp133 in BFR, but it is possible that they function to facilitate the formation and/or decay of the Tyr25 radical, and one possibility is that these residues function in transferring the radical away from the ferroxidase site, enabling the site to return to its resting state (Figure 4).

In summary, we have shown that three aromatic residues surrounding the EcBFR diiron site are crucial for mineralization, consistent with a role in electron transfer from Fe^2+^ in the cavity to the ferroxidase site, through a mechanism that involves transient formation of a Tyr25 radical. Several questions remain to be answered, particularly concerning formation and decay of the radical and the roles of Tyr58 and Trp133 in this, and the nature of the diiron species that oxidizes Tyr25.

## Supplementary material

As a service to our authors and readers, this journal provides supporting information supplied by the authors. Such materials are peer reviewed and may be re-organized for online delivery, but are not copy-edited or typeset. Technical support issues arising from supporting information (other than missing files) should be addressed to the authors.
