# Supplementary material for: Three Aromatic Residues are Required for Electron Transfer during Iron Mineralization in Bacterioferritin
Source: Angew Chem Int Ed Engl. 2015 Oct 16;54(49):14763–7. doi: 10.1002/anie.201507486 (PMC4691338; doi:10.1002/anie.201507486)
Supplement: Supplementary file 1 [file anie0054-14763-SD1.pdf]

Angewandte Chemie
Eine Zeitschrift der Gesellschaft Deutscher Chemiker

Supporting Information

Three Aromatic Residues are Required for Electron Transfer during Iron Mineralization in Bacterioferritin

*Justin M. Bradley, Dimitri A. Svistunenko, Tamara L. Lawson, Andrew M. Hemmings, Geoffrey R. Moore, and Nick E. Le Brun**

anie_201507486_sm_miscellaneous_information.pdf

Experimental Methods

Site-directed mutagenesis and protein purification:

Escherichia coli strains JM109 and BL21(DE3) (Promega) were used for site-directed mutagenesis and expression, respectively, as previously described.[1] To generate Y25F BFR, site directed mutagenesis was carried out using a whole-plasmid method with GCAATCAATCAGTTTTTCTCCATGCC and its complementary sequence as primers and pALN1 as template.[1] The resulting plasmid (pTLN7) was cut with NdeI and EcoRI and the fragment was cloned into pET21a, giving pTLN9. Mutations were confirmed by sequencing (MWG Biotech, Ebsberg, Germany). Variants Y10F, Y45F, Y58F, Y107F, Y114F and Y149F were all purchased from GenScript (New Jersey, USA).

Wild-type and variant BFR proteins including the Tyr variants above and the previously generated W133F, W35F[2] and H46A[1] variants, were prepared as previously described,[3] except that expression was induced with 10 µM IPTG rather than 1 mM. Non-heme iron was removed by treatment with sodium dithionite and bipyridyl, as previously described.[4] The concentrations of BFR proteins were determined using per subunit $\varepsilon_{280\ nm}$ values that were either already known, or determined either by amino acid analysis (Alta Bioscences, University of Birmingham) or by protein quantification using the BCA assay (Sigma). The two methods were found to yield values in agreement to within 3%. Per subunit $\varepsilon_{280\ nm}$ values were: 33,000 (wild-type and H46A),[1, 5] 22,300 (W35F); 23,375 (W133F);[2] 25,585 (Y25F); 24,200 (Y10F); 27,100 (Y45F); 24,600 (Y58F); 28,000 (Y107F); 28,500 (Y114F); and, 25,600 (Y149F), all in units of $M^{-1}\ cm^{-1}$. Heme content of proteins was determined following non-heme iron removal using the heme Soret absorbance intensity ($\varepsilon_{418\ nm}$ = 107,000 $M^{-1}\ cm^{-1}$),[6] and found to be 1.0-1.5 heme/BFR for all variants. Note that the lack of heme does not significantly affect the rate of mineralization at low to mid-iron loadings.[7]

Kinetic methods:

Kinetic measurements of changes in absorption at 340 nm after the addition of Fe^{2+} (as ferrous ammonium sulfate) to apo-BFR proteins were made using either a conventional U.V.-visible spectrophotometer (Perkin-Elmer λ35, Hitachi U2900 or Jasco V550), for which additions to the sample were made using a micro-syringe (Hamilton), or by using a stopped-flow apparatus (Applied Photophysics Bio-Sequential DX.17MV spectrophotometer with a 1 cm path length cell) with the BFR solution in one syringe and the metal ion solution in another. Oxidation of Fe^{2+} to Fe^{3+} was monitored at 340 nm.[8] Rates of Fe^{2+} oxidation following this initial ferroxidase center reaction, which report on core formation, were calculated from starting, linear increases in $A_{340\ nm}$ per unit time. Because $\varepsilon_{340\ nm}$ values could not be assumed to be constant between samples, values of $\Delta A_{340\ nm}$ min^{-1} values were converted to Fe^{2+} oxidised per min (µM min^{-1}).[3]

EPR spectroscopy:

Spectra were recorded at 10 K on a Bruker EMX (X-band) EPR spectrometer equipped with an Oxford Instruments liquid helium system and a spherical high-quality ER 4122 SP 9703 Bruker resonator. Protein samples in EPR tubes were mixed with the appropriate volume of a 25 mM stock Fe^{2+} solution and frozen ≈10 s thereafter by plunging the tubes into methanol cooled with solid CO_2. Samples frozen at times < 10 s were prepared by mixing equal volumes

of a 16.66 µM protein solution (400 µM in subunit monomer) and a 1.2 mM Fe^{2+} solution in an Update Instrument 715 Syringe Ram Controller (Madison, WI) and ejecting the mixture on to the surface of a rapidly rotating aluminium disk kept at liquid nitrogen temperature, as described elsewhere.[9] Final protein concentration was 8.33 µM (200 µM in monomer) in all cases. Free radical concentration was determined by double integration of the EPR spectra using a Cu^{2+} concentration standard. The tyrosyl radical EPR spectrum was simulated by SimPow6[10] using parameters derived from the Tyrosyl Radical Spectra Simulation Algorithm (TRSSA).[11] The input parameters for TRSSA were optimized manually to give the best fit between the simulated and observed spectra.

PDB structure files analysis:

The analysis of the rotational conformation of all tyrosines in six PDB structure files of wild type E. coli BFR (3E1J: apo-BFR; 3E1L: phosphate-soaked apo-BFR; 3E1M: apo-BFR aerobically soaked in Fe^{2+} for 2.5 min; 3E1N: apo-BFR aerobically soaked in Fe^{2+} for 65 min; 3E1O: apo-BFR aerobically soaked in Zn^{2+}; 3E1P: apo-BFR aerobically soaked in Zn^{2+} followed by Fe^{2+})[1] was performed using an on-line database.[12] This allowed the tyrosines within each structure to be ranked according to the similarity of their ring rotation angle θ to the values found from the simulations of EPR spectra.

Structural methods:

Crystals of Y45F, Y114F, Y149F variant proteins were prepared according to the previously published procedure.[1] Proteins were crystalized using the sitting drop method. Briefly, samples were exchanged into 20 mM Mes pH 6.5 (protein concentration 10 mg ml^{-1}) and 2 µl drops equilibrated with an equal volume of well solution (1.6 M ammonium sulfate, 0.1 M sodium citrate pH 5.0) prior to seeding with microcrystals of wild type protein. Crystals appeared in 3-4 days and grew to optimum size in 10-12 weeks. Crystals could be cryoprotected by transfer to a stabilization solution containing 30% (v/v) glycerol. X-ray diffraction data was collected on stations I03 (Y45F), I04 (Y149F) and I04-1 (Y114F) of the Diamond Light Source and processed using Xia2.[13] Molecular replacement used the high resolution structure of WT BFR (pdb entry 2Y3Q) as the search model and was carried out using phenix phaser MR.[14] Model refinement employed iterative cycles using phenix.refine[15] and manual correction using COOT.[16] Data collection and refinement statistics are given in Table S1.

Supporting Data

Supporting Tables

Table S1. Crystal structure data collection and refinement statistics. Values in parentheses are those for the highest resolution bin of the diffraction data.

	Y45F	Y114F	Y149F
PDB Entry	4XKS	4XKU	4XKT
**Data collection**[a]			
Wavelength, Å	0.9763	0.9800	0.9200
Space group	$C222_1$	$P\ 4_22_12$	$P\ 4_22_12$
Cell parameters a , b , c (Å)	127.3, 196.0, 201.9	207.6, 207.6, 142.6	208.5, 208.5, 142.9
Resolution limits, Å	106.8 – 1.57 (1.61 – 1.57)	102.27 – 1.77 (1.83 – 1.77)	31.74-1.87 (1.87-1.82)
R-calc	0.088 (0.560)	0.093 (0.665)	0.129 (0.767)
(I)/σ(I)	15.6 (2.5)	12.6 (2.5)	20.3 (5.5)
Completeness, %	98.4 (85.6)	96.5 (98.5)	99.8 (99.9)
Multiplicity	9.7 (5.1)	6.8 (6.8)	27.0(27.4)
Overall temperature factor, $Å^2$	14.7	15.6	14.9
**Refinement Statistics**			
Protein monomers per asymmetric unit	12	12	12
Total atoms	18749	19042	19321
Water molecules	2838	2987	2769
R-work, %	14.8	18.8	16.6
R-free, %	17.2	21.7	20.5
Ramachandran Analysis, %			
Most favoured	100	99.9	99.7
Outliers	0	0.0	0.0
RMS deviations			
Bonds, Å	0.005	0.006	0.006
Angles, °	0.919	0.898	0.860
Planes, Å	0.003	0.003	0.004
Mean Atomic B-value, $Å^2$	18.0	11.0	14.0

Table S2. Tyr radical EPR spectrum simulation parameters. Parameters were generated by TRSSA[11] for the input of ɵ = -17.4° and ρ_{c1} = 0.408

**g values**	g_x	g_y	g_z
	2.00696	2.00419	2.00221
**Hyperfine coupling parameters (MHz)***	$A^{\beta 1}cc$	$A^{\beta 1}bb$	$A^{\beta 1}aa$
	59.60	53.26	53.26
	$A^{\beta 2}cc$	$A^{\beta 2}bb$	$A^{\beta}aa$
	35.43	31.39	31.39
**linewidths (G)**	ΔH_x	ΔH_y	ΔH_z
	7.70	5.97	5.68

* all other hyperfine coupling parameters and the Euler angles defining the relative orientation of the hyperfine- and g- matrices were assumed constant during the refinement.

Supporting Figures

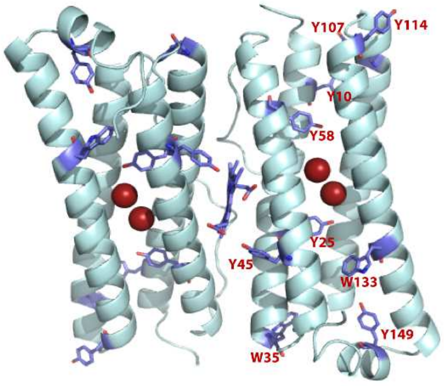

Figure S1. Trp and Tyr residues of *E. coli* BFR. View of a subunit dimer (12 such dimers make up the 24mer) with the irons of the ferroxidase centers shown as brick red spheres and the heme group bound at an inter-subunit site. Trp and Tyr residues are shown in sticks representation Generated using PyMol with PDB file 3E1M.[17]

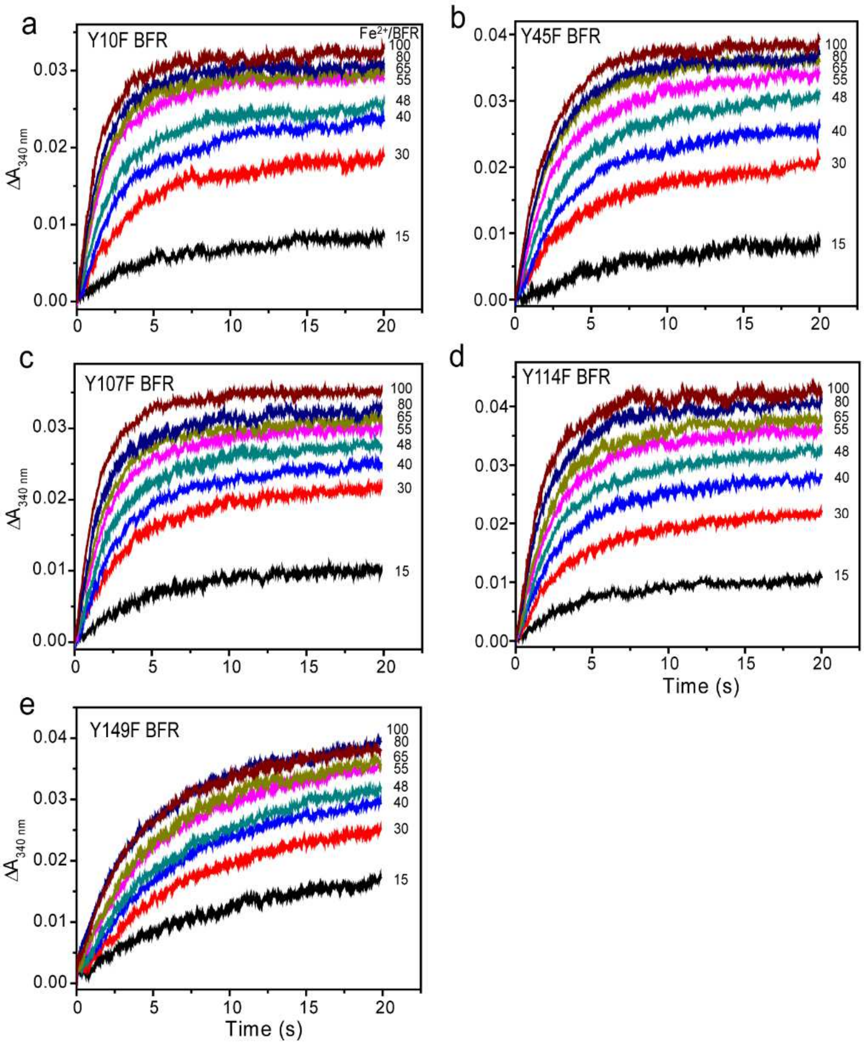

Figure S2. Kinetics of the initial ferroxidase center reaction in BFR aromatic residue variants. $\Delta A_{340\ nm}$ measured by stopped-flow during the first 20 s following additions of 0 – 100 Fe^{2+} per (a) Y10F, (b) Y45F, (c) Y107F, (d) Y114F and (e) Y149F BFRs. The proteins (1 μM in 0.1 M MES pH 6.5) were mixed 1:1 with appropriate iron solutions. Temperature was 25 ºC and path length 1 cm.

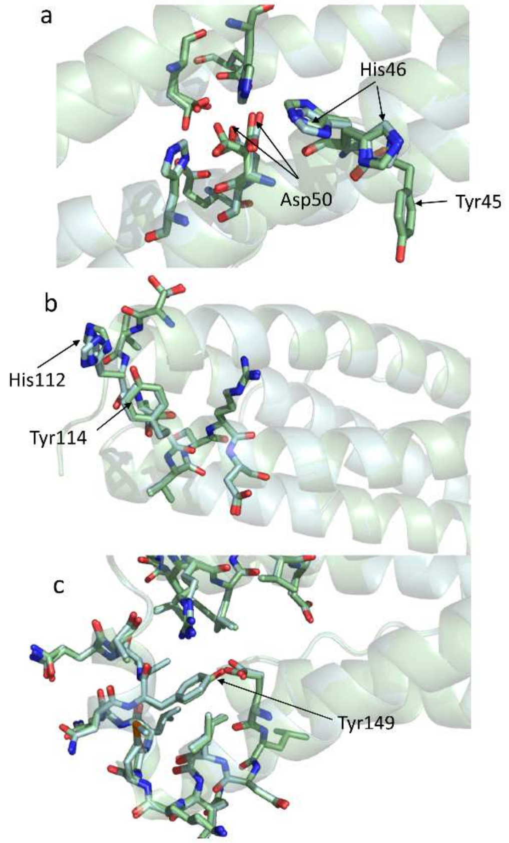

Figure S3. X-ray structures of Tyr variants. Structures of (a) Y45F, (b) Y114F and (c) Y149F BFRs, all in cyan overlaid with the wild-type BFR structure in grey. Panel (A) also details the positions of the ferroxidase centre ligands that are unaffected by the mutation. The conformations of the Fe_{IS} ligands His46 and Asp50 do, however, show small changes in the Y45F variant, consistent with the ~50% decrease in activity observed for that variant (see Fig. 2a of the main paper). Wild-type BFR and Y114F structures are identical other than in the side chain of the mutated residue and the orientation of His112 which lies on an unstructured loop. The Y149F mutation causes no significant perturbation of the protein structure.

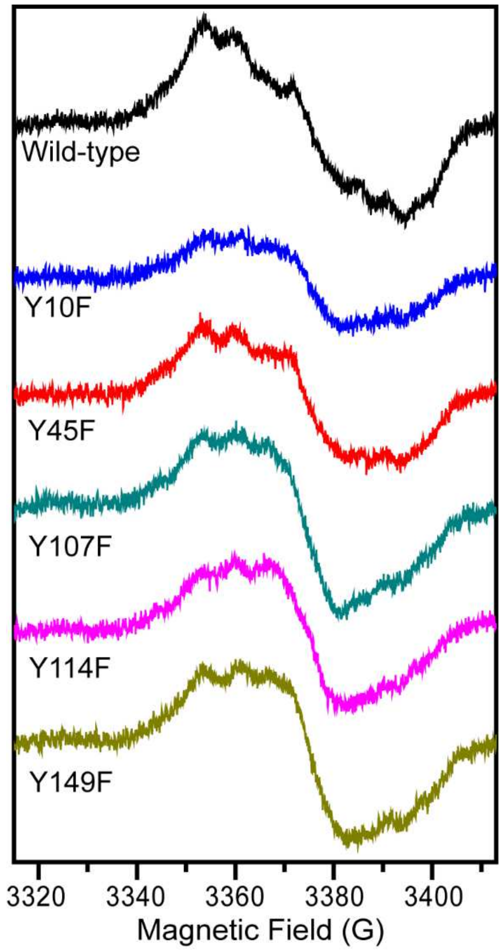

Figure S4. Trp and Tyr residues of *E. coli* BFR and analysis of tyrosyl radical in wild-type and variant BFRs. EPR spectra following addition of 72 Fe^{2+}, per protein to wild-type BFR and variants (8.3 µM in 0.1 M MES pH 6.5), as indicated. For all spectra, solutions were frozen 10 s after iron addition. The EPR spectra were obtained at 10 K at the instrumental conditions specified in the legend of Fig. 3 in the main paper.

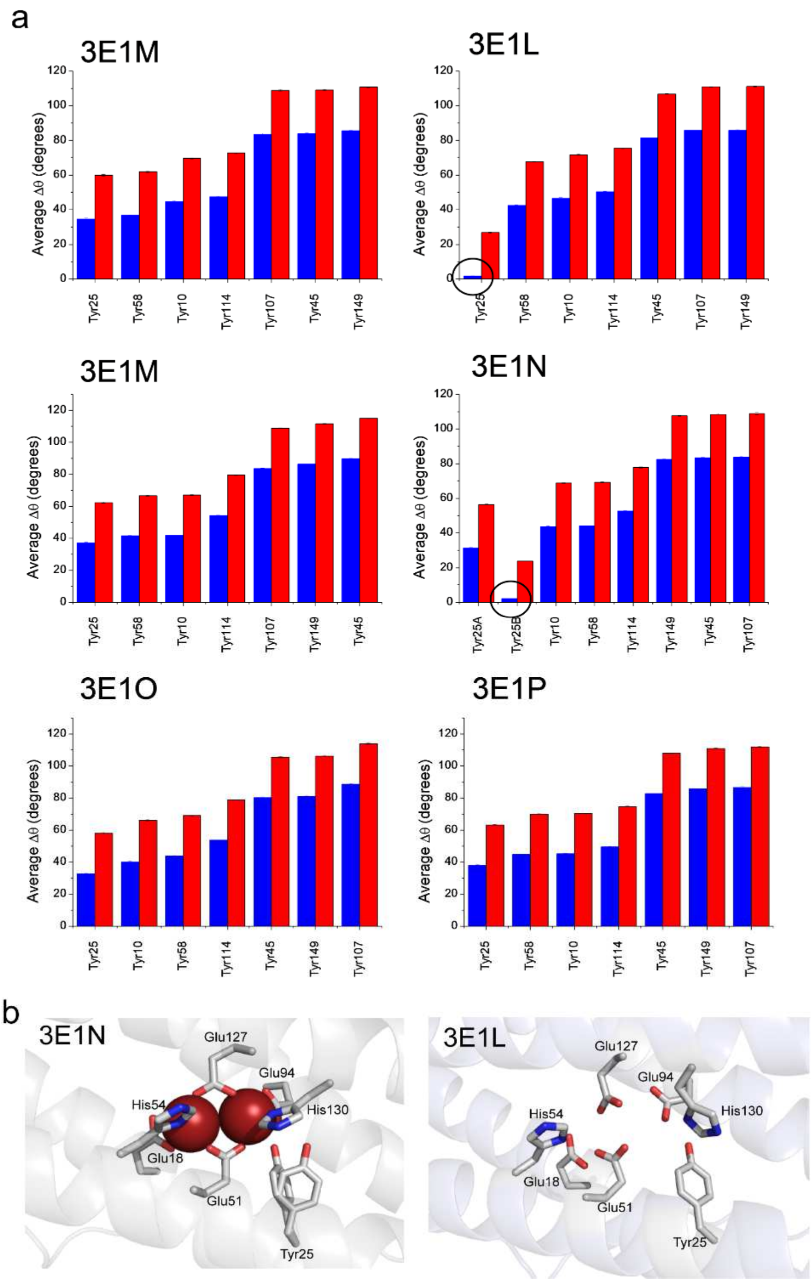

Figure S5. Analysis of BFR tyrosine ring rotational conformations. (a) Differences between the observed ring rotation angles of the seven tyrosine side chains present in BFR and those predicted by from simulation of the EPR spectrum; -17.4º (blue) and -42.6º (red). Observed orientations are averaged over the 12 homo-monomers of the asymmetric unit and the standard deviation indicated. Note that two conformations of Tyr25 are observed in the iron soaked structure 3E1N.[17] Tyr residues with conformations matching that predicted are highlighted with a circle. (b) Left hand panel, structure of iron-bound *E. coli* BFR (from pdb 3E1N) showing two conformations for Tyr25.[17] The

one with the ring closer to the diiron center is predicted to give rise to the observed Tyr radical signal. Right panel, strucure of *E. coli* apo-BFR containing Tyr in a similar ‘Tyr radical’ conformation (pdb 3E1L).[17]

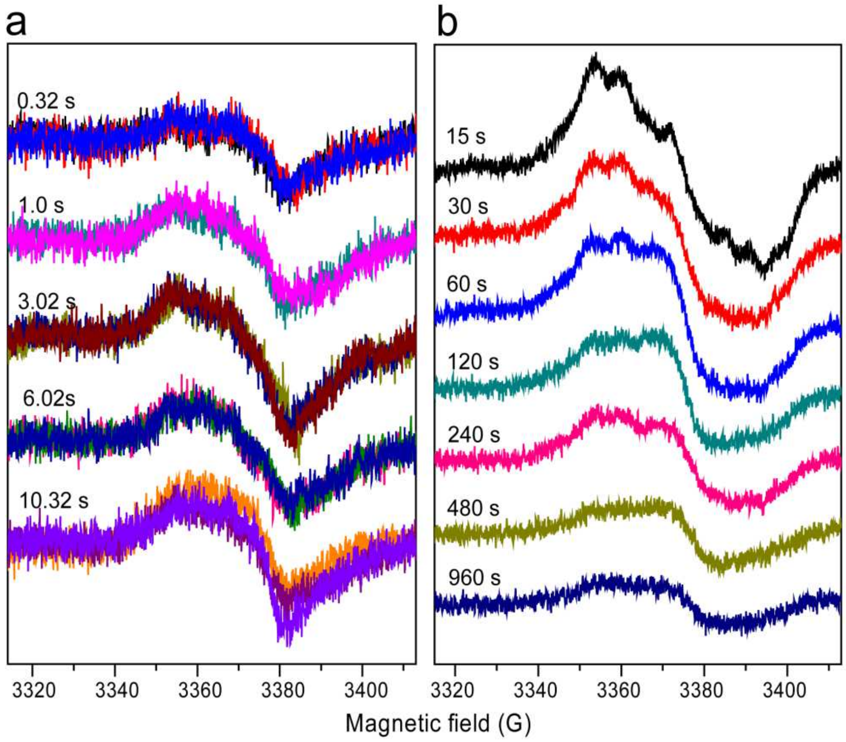

Figure S6. Time course EPR studies of Tyr25 radical formation and decay. (a) EPR spectra of wild type BFR rapidly freeze-quenched at increasing time (as indicated) after addition of 72 Fe^{2+} per protein. Each time point is represented by three overlaid spectra from similar independent freeze-quenched samples (with exception of the group "1 s" for which two spectra are reported). (b) EPR spectra of wild type BFR frozen normally at variable time (as indicated) after addition of 72 Fe^{2+}. BFR was 8.3 µM in 0.1 M MES pH 6.5. Instrumental conditions were as in the legend of Fig. 3 of the main paper.

Supporting References

[1] A. Crow, T. L. Lawson, A. Lewin, G. R. Moore, N. E. Le Brun, *J. Am. Chem. Soc.* **2009**, *131*, 6808-6813.

[2] T. L. Lawson, A. Crow, A. Lewin, S. Yasmin, G. R. Moore, N. E. Le Brun, *Biochemistry* **2009**, *48*, 9031-9039.

[3] S. Baaghil, A. Lewin, G. R. Moore, N. E. Le Brun, *Biochemistry* **2003**, *42*, 14047-14056.

[4] E. R. Bauminger, P. M. Harrison, D. Hechel, I. Nowik, A. Treffry, *Biochim. Biophys. Acta* **1991**, *1118*, 48-58.

[5] X. Yang, N. E. Le Brun, A. J. Thomson, C. R. Moore, N. D. Chasteen, *Biochemistry* **2000**, *39*, 4915-4923.

[6] M. R. Cheesman, N. E. Le Brun, F. H. A. Kadir, A. J. Thomson, G. R. Moore, S. C. Andrews, J. R. Guest, P. M. Harrison, J. M. A. Smith, S. J. Yewdall, *Biochem. J.* **1993**, *292*, 47-56.

[7] a) S. C. Andrews, N. E. Le Brun, V. Barynin, A. J. Thomson, G. R. Moore, J. R. Guest, P. M. Harrison, *J. Biol. Chem.* **1995**, *270*, 23268-23274; b) S. G. Wong, R. Abdulqadir, N. E. Le Brun, G. R. Moore, A. G. Mauk, *Biochem. J.* **2012**, *444*, 553-560.

[8] N. E. Le Brun, M. T. Wilson, S. C. Andrews, J. R. Guest, P. M. Harrison, A. J. Thomson, G. R. Moore, *FEBS Lett.* **1993**, *333*, 197-202.

[9] M. K. Thompson, S. Franzen, R. A. Ghiladi, B. J. Reeder, D. A. Svistunenko, *J. Am. Chem. Soc.* **2010**, *132*, 17501-17510.

[10] M. J. Nilges, K. Mattson, R. L. Belford, *Bio. Magn. Res.* **2007**, *27*, 261-281.

[11] D. A. Svistunenko, C. E. Cooper, *Biophys. J.* **2004**, *87*, 582-595.

[12] D. A. Svistunenko, **2004**. <http://privatewww.essex.ac.uk/~svist/lev1/tyrdb/home.shtml>.

[13] G. Winter, *J. Appl. Crystallogr.* **2010**, *43*, 186-190.

[14] A. J. McCoy, R. W. Grosse-Kunstleve, P. D. Adams, M. D. Winn, L. C. Storoni, R. J. Read, *J. Appl. Crystallogr.* **2007**, *40*, 658-674.

[15] P. V. Afonine, R. W. Grosse-Kunstleve, N. Echols, J. J. Headd, N. W. Moriarty, M. Mustyakimov, T. C. Terwilliger, A. Urzhumtsev, P. H. Zwart, P. D. Adams, *Acta Crystallogr. D, Biol. Crystallogr.* **2012**, *68*, 352-367.

[16] P. Emsley, K. Cowtan, *Acta Crystallogr. D, Biol. Crystallogr.* **2004**, *60*, 2126-2132.

[17] A. Crow, T. L. Lawson, A. Lewin, G. R. Moore, N. E. Le Brun, *J. Am. Chem. Soc.* **2009**, *131*, 6808-6813.
